# 

**Published:** 1961-07

**Authors:** 


### MEDICAL JOURNAL OF THE SOUTH-WEST

*(Incorporating the Bristol Medico-Chirurgical Journal)*

Established 1883

VOL. 76 (iii) July 1961 NO. 281

### *Contents*

SOME LESSER-KNOWN ACTIVITIES OF THE GENERAL MEDICAL COUNCIL . . . . . . *Professor R. J. Brocklehurst* 75

REFLECTIONS ON THE INDICATIONS FOR PROSTATECTOMY *J. P. Mitchell* 85

A CASE OF UNILATERAL INVOLUNTARY MOVEMENTS TREATED WITH PROCAINE AMIDE . . . . . *W. Cullen and A. Leitch* 92

A COAGULATION DEFECT IN AMYLOIDOSIS *I. D. Fraser and M. R. Wills* 97

NOTES AND NEWS . . . . . . . . 101

APPOINTMENTS . . . . . . . . . 101

*PUBLISHED QUARTERLY*

*ANNUAL SUBSCRIPTION* **16/-** *POST FREE*

PUBLISHED UNDER THE AUSPICES OF THE

RISTOL MEDICO-CHIRURGICAL SOCIETY

### *Editorial Committee*

Dr. J. E. Cates, Department of Medicine, Bristol Royal Infirmary. *Editor.*

Dr. N. J. Brown, Southmead Hospital, Bristol. *Assistant Editor.*

Dr. A. B. Raper, Department of Pathology, Bristol Royal Infirmary. *Assistant Editor.*

Dr. J. Macrae, Ham Green Hospital.

Dr. R. C. Wofinden, Central Clinic, Bristol.

Mr. A. E. S. Roberts, Medical Library, University of Bristol. *Secretary.*

### *Local Correspondents*

*Bath* . . Mr. A. D. Bateman, 3 The Circus, Bath.

*Exeter* . . Dr. L. N. Jackson, Mount Jocelyn, Crediton, Devon.

*Gloucester* . Dr. R. F. Jarrett, 9 College Green, Gloucester.

*Plymouth* . Dr. C. R. Croft, Lexden, Hartley Av., Plymouth.

*Taunton* . Dr. M. McAlley, 1 The Crescent, Taunton.

*Torquay* . Dr. A. Robinson Thomas, 20 Devon Square, Newton Abbot, Devon.

*Truro* . . Dr. F. D. M. Hocking, St. Andrews, Perranporth, Cornwall.

*Yeovil* . . Mr. J. A. Vere Nicoll, Knapp House, Yeovil, Somerset.

### NOTICE TO CONTRIBUTORS

Original articles are invited, provided they have not been published elsewhere. Notes of forthcoming events, meetings held, degrees conferred, staff appointments, and other ocal news are welcomed. The editorial committee reserves the right to make literary corrections.

Illustrations should always be supplied ready for reproduction. All re-touchings or other operations required will be charged to the author. Line drawings should be drawn in Indian ink. We regret that we must ask authors to pay for making blocks, if this is necessary.

J. W. ARROWSMITH LTD., WINTERSTOKE ROAD,

BRISTOL.

### Notes and News

Obituary: V. B. Green-Armytage, M.D. (Bristol), F.R.C.P., F.R.C.S., F.R.C.O.G.

### Appointments

| *Name* | *Appointment* | |
|---|---|---|
| Beeson, B. B., M.B. (Bristol) M.R.C.P.E. | Consultant Pathologist. | Lancaster and Kendal and Lancaster Moor Hospital Group. |
| Dunnill, M. S., M.D. (Bristol) M.R.C.P. | Consultant Pathologist. | Radcliffe Infirmary, Oxford. |
| Pengelly, C. D. R., M.D. (Bristol), M.R.C.P. | Consultant Physician. | North and Mid-Cheshire Hospital Group. |

### *SOUTH WESTERN REGIONAL HOSPITAL BOARD JANUARY TO MARCH 1961*

### *BRISTOL*

| *Name* | *Appointment* | *Formerly* |
|---|---|---|
| Hill, B. W., M.B., Ch.B.(Bristol) | Clinical Assistant in Dermatology, Cossham Memorial Hospital (one session) re-appointed. | In general practice in Bristol. |
| Wethered, H. H., B.A., M.B., B.Ch.(Oxford). | Clinical Assistant in Dermatology, Southmead Hospital (two sessions re-appointed. | In general practice in Bristol. |
| Alford, R. M., M.R.C.S., L.R.C.P.(Lond.). | Clinical Assistants in Obstetrics, Weston-Super-Mare General Hospital. | In general practice in Weston-Super-Mare. |
| Nott-Bower, T. M., M.R.C.S.(Eng.), L.R.C.P. (Lond.), D.Obst.R.C.O.G. | Clinical Assistants in Obstetrics Weston-Super-Mare General Hospital. | In general practice in Weston-Super-Mare. |
| Bennett, P. M. J., B.A. (Cantab.), M.B., B.Chir. | Medical Regsitrar, Cossham–Frenchay Group of Hospitals. | (S.H.O.), Frenchay Hospital. |
| Burns, B. H., M.B., Ch.B. (Bristol), D.Obst.R.C.O.G. | Registrar in Phychiatry, Glenside and Barrow Group of Hospitals. | Captain in the Army (completing National Service at Tidworth Military Hosp.). |
| James, B., B.Sc., M.B., B.Ch.(Wales). | Registrar in Phychiatry, Glenside and Barrow Group of Hospitals. | Morgannwg Hospital, Bridgend, Glam. |
| Johnson, J. K. M.B., Ch.B. (Manchester), D.Obst.R.C.O.G. | Registrar in Pathology, Frenchay Hospital. | S.H.O. in Pathology, Frenchay Hospital. |
| Mehendale, C. V. M.B., B.S., B.Sc.(Anatomy), M.S. (Gen. Surgery)(Bombay). | Registrar in Plastic Surgery, Frenchay Hospital. | Locum Registrar in Plastic Surgery, Frenchay Hospital. |
| Nour-Eldin, F., M.B., B.Ch.(Cairo), Ph.D.(Manchester), L.M.S.S.A.(Lond.). | Registrar in Pathology, Southmead Hospital. | J.H.M.O., Blood Transfusion Service, Brentwood. |

### *BATH*

| | | |
|---|---|---|
| Berry, G., M.B., Ch.B.(Leeds), D.Obst.R.C.O.G., D.A.(Lond.). | Anaesthetic Registrar, Bath Group of Hospitals. | Asst. Professor of Anesthesiology, Vanderbilt University, U.S.A. |

| *Name* | *Appointment* | *Formerly* |
|---|---|---|
| COBBAM, I., M.A., B.M., B.CH.(OXON). | Anaesthetic Registrar, Bath Group of Hospitals. | Resident Anaesthetist, Royal Berkshire Hospital, Reading |
| MANUDANE, G. S., M.B., B.S., D.O.R.L. (BOMBAY), D.L.O.(LOND.). | Registrar in E.N.T. Surgery, Bath Group of Hospitals. | Locum E.N.T. Registrar, Hillingdon Hospital, Uxbridge, Middx. |
| STEWART, D. J., M.B., CH.B.(GLASGOW). | Re-appointed for two years as Clinical Assistant in Orthopaedics and Traumatic Surgery, Royal United Hospital, Bath. | In general practice in Bath. |
| | *NORTH GLOUCESTERSHIRE* | |
| CROUCH, H. E., M.B., B.CH., B.A.O.(BELFAST). | Re-appointed for two years as Clinical Assistant in Dermatology, Stroud Hospital and Gloucester Royal Hospital, Gloucester | — |
| SEN GUPTA, P. C., M.B., B.S.(CALCUTTA), D.G.O.(CALCUTTA), M.R.C.O.G. | Registrar in Obstetrics and Gynaecology, Gloucestershire Royal and City Maternity Hospitals, Gloucester. Re-appointed. | Registrar in Obstetrics and Gynecology, Gloucester. |
| | *SOUTH SOMERSET* | |
| FOX, P. P., M.B., CH.B. (LIVERPOOL), D.P.H. | G.P. Specialist in Venereology, Yeovil General Hospital, Yeovil. (one session per wk.). | Medical Officer of Health, Yeovil. |
| | *DEVON AND CORNWALL* | |
| LLOYD-DAVIES, D. G., F.R.C.S.(ED.), D.L.O.(ENG.), F.A.C.S., M.D.(MANITOBA), L.M.C.C.(CANADA). | Consultant E.N.T. Surgeon, North Devon Infirmary, Barnstaple. | Consultant E.N.T. Surgeon Dudley Road Hospital, Birmingham. |
| SHEERS, G., M.A., M.B., B.CHIR., M.D.(CANTAB.). | Temporary Consultant Chest Physician, Plymouth Clinical Area for a period of two years. | S.H.M.O. Mass Radiography Centre, Devonport, Plymouth. |
| CARDALE, J. D., M.B., CH.B.(BRISTOL). | G.P. Surgeon in Orthopaedic Surgery at Lydney and District Hospital for one session weekly. Re-appointed for a period of two years. | In general practice in Gloucester. |
| DAVIDSON, C. M., M.B., CH.B.(GLASGOW), F.R.C.S.(EDIN.). | Senior Surgical Registrar, Royal Devon and Exeter Hospital (Joint appt. with United Bristol Hospitals). | Surgical Registrar, Royal Infirmary, Glasgow. |
| GANDHI, D. A., M.B., B.S., (BARODA), D.T.M. &. H. (ENG.). | Medical Registrar, Royal Cornwall Infirmary, Truro. | Locum Registrar in Geriatrics, St. Matthew's Hospital, London. |
| SOUTHWOOD, R. T., M.B., B.S.(ADELAID), F.R.C.S. | Registrar in Orthopaedic and Traumatic Surgery, Torbay Hospital/ Princess Elizabeth Orthopaedic Hospital, Exeter. | Surgical Registrar, Warwick Hospital Warwick. |

